# Changes in brain activation in breast cancer patients depend on cognitive domain and treatment type

**DOI:** 10.1371/journal.pone.0171724

**Published:** 2017-03-07

**Authors:** Sanne Menning, Michiel B. de Ruiter, Dick J. Veltman, Willem Boogerd, Hester S. A. Oldenburg, Liesbeth Reneman, Sanne B. Schagen

**Affiliations:** 1 Division of Psychosocial Research and Epidemiology, Netherlands Cancer Institute, Amsterdam, The Netherlands; 2 Department of Radiology, Academic Medical Center, University of Amsterdam, Amsterdam, The Netherlands; 3 Department of Psychiatry, VU University Medical Center, Amsterdam, The Netherlands; 4 Department of Neuro-Oncology, Netherlands Cancer Institute, Amsterdam, The Netherlands; 5 Department of Surgical Oncology, Netherlands Cancer Institute, Amsterdam, The Netherlands; Universitair Medisch Centrum Groningen, NETHERLANDS

## Abstract

**Background:**

Cognitive problems in breast cancer patients are common after systemic treatment, particularly chemotherapy. An increasing number of fMRI studies show altered brain activation in breast cancer patients after treatment, suggestive of neurotoxicity. Previous prospective fMRI studies administered a single cognitive task. The current study employed two task paradigms to evaluate whether treatment-induced changes depend on the probed cognitive domain.

**Methods:**

Participants were breast cancer patients scheduled to receive systemic treatment (anthracycline-based chemotherapy +/- endocrine treatment, n = 28), or no systemic treatment (n = 24) and no-cancer controls (n = 31). Assessment took place before adjuvant treatment and six months after chemotherapy, or at similar intervals. Blood oxygen level dependent (BOLD) activation and performance were measured during an executive functioning task and an episodic memory task. Group-by-time interactions were analyzed using a flexible factorial design.

**Results:**

Task performance did not differ between patient groups and did not change over time. Breast cancer patients who received systemic treatment, however, showed increased parietal activation compared to baseline with increasing executive functioning task load compared to breast cancer patients who did not receive systemic treatment. This hyperactivation was accompanied by worse physical functioning, higher levels of fatigue and more cognitive complaints. In contrast, in breast cancer patients who did not receive systemic treatment, parietal activation normalized over time compared to the other two groups.

**Conclusions:**

Parietal hyperactivation after systemic treatment in the context of stable levels of executive task performance is compatible with a compensatory processing account of hyperactivation or maintain adequate performance levels. This over-recruitment of brain regions depends on the probed cognitive domain and may represent a response to decreased neural integrity after systemic treatment. Overall these results suggest different neurobehavioral trajectories in breast cancer patients depending on treatment type.

## Introduction

Treatment-related cognitive impairment in patients with cancer outside the central nervous system (CNS) has observed in a large number of neuropsychological studies, mostly focusing on breast cancer (BC) patients [1]. Many neuropsychological studies show that adjuvant chemotherapy is associated with increased rates of cognitive impairment, while preclinical studies report neurotoxicity of many chemotherapeutic agents [2,3]. Endocrine treatment is another frequently prescribed type of adjuvant systemic treatment that might also contribute to cognitive problems, although few studies have specifically focused on its cognitive side effects [4–6]. Although prevalence and severity of impairments vary between studies, commonly affected cognitive domains are memory and executive function [7]. However, the underlying neural mechanisms of these cognitive impairments remain largely unknown. Preclinical studies have demonstrated increased apoptosis in healthy proliferating cells in the central nervous system, as well as damage to neural precursor cells [3]. Also, functional MRI (fMRI) has been employed to study the effects of cancer and treatment on brain activation in non-CNS patients [8].

Chemotherapy-associated changes in regional brain activation, by means of fMRI, have been described in three longitudinal studies [9–11]. After surgery but before adjuvant treatment, McDonald et al. [9] reported frontal and parietal hyperactivation during working memory performance in BC patients, either scheduled to receive chemotherapy (CHT+) or not requiring chemotherapy (CHT-) compared to no-cancer controls (NC). Further, right inferior frontal activation was higher in CHT+ compared with CHT-. One month after completion of chemotherapy, or at a similar time point, both CHT+ and CHT- showed decreased activation of the inferior frontal cortex compared to NC, accompanied by a non-significant drop in task performance in the CHT+ group. This pattern was not found in the other groups. The authors hypothesized that CHT+ had not been able to maintain performance through compensatory hyperactivation at one month following treatment. At one-year follow-up, regional brain activation in patients had returned to pretreatment levels, with CHT+ patients additionally showing hyperactivation of bilateral middle frontal gyri compared to CHT-.

Lopez-Zunini et al. [10] studied breast cancer patients receiving chemotherapy and NCs during a verbal memory recall task. Between-group analyses before chemotherapy showed hyperactivation of the right anterior cingulate cortex (ACC) in patients compared to NC. One month after chemotherapy, hypoactivation of the right insula and middle temporal gyrus was found in CHT+ compared to NC. CHT+ showed decreased activation in bilateral insula and left orbitofrontal cortex at T2 compared to T1, while NCs showed no changes. Changes over time in CHT+ were no longer significant when ‘days since surgery’ was taken into account. The authors also studied the effects of multiple psychosocial factors on regional brain activation and found that anxiety, depression and fatigue modulated the differences between patients and controls. Verbal recall task performance showed no significant group-by-time interaction.

Deprez et al. [11] reported no group differences in brain activation during multitasking between CHT+, CHT- and NC before chemotherapy. Within-group analyses showed a decrease in activation in the left ACC and the intraparietal sulcus four to six months after treatment in the CHT+ group, whereas no changes over time were found in CHT- and NC. Group-by-time interaction analysis showed the decrease in activation in the ACC in the CHT+ group to be significantly different from activation in the NC group. No significant differences in performance were found. However, the decreased activation in CHT+ patients was related to a higher frequency of cognitive complaints.

These studies show divergent patterns of brain activation over time for CHT+, CHT- and NC. Differences in location as well as direction of changes in brain activation following treatment in BC patients were found. This could be due to the employment of different cognitive tasks, relying on distinct brain areas. Several factors have been found to be related to these differences in brain activation. Deprez et al. [11] reported correlations between BOLD signal and self-reported cognitive problems. Lopez-Zunini et al. [10] showed different psychosocial factors to modulate the changes over time. Prior to treatment, our group found differences in brain activation between BC patients and controls to be driven by fatigue [12].

In the present prospective study, BC patients receiving systemic treatment (anthracycline-based chemotherapy +/- endocrine treatment = BC+SYST) were compared to women without cancer (NC) as well as to BC patients not requiring systemic treatment (BC), according to recommendations of the International Cognition and Cancer Task Force (ICCTF) [13]. This allowed us to separately study the effects of cancer and systemic treatment on brain activation. Two fMRI tasks were employed to cover the cognitive domains of executive function and memory, which have previously been reported to be affected following cancer treatment [1,2,7,14,15]. This allowed us to examine the effects of cancer and cancer treatment on brain activation in multiple cognitive domains, instead of being restricted to one specific task. In addition, we evaluated the relation between brain activation and patient-reported outcomes. We hypothesized that patients receiving systemic treatment would show altered brain activation compared to BC patients not requiring systemic treatment and to no-cancer controls. In addition, we expected baseline measures of fatigue to be related to changes in brain activation in both patient groups.

## Materials and methods

### Participants

Participants were patients with BC, who were scheduled to receive adjuvant anthracycline-based chemotherapy with or without endocrine treatment (BC+SYST), or who did not require systemic treatment (BC), and age-matched no-cancer controls (NC). Participants were eligible if they met the following criteria: female, under the age of 70 years, sufficient command of the Dutch language, no previous malignancies. Additionally, patients had to have a diagnosis of primary breast cancer, no distant metastases and no other treatment than surgery at the time of baseline assessment. Patients scheduled to receive trastuzumab following chemotherapy were not eligible because of the longer treatment duration compared to patients receiving chemotherapy without trastuzumab. NCs were recruited via participants, as well as through advertisements for personnel in the participating hospitals.

The study was approved by the Institutional Review Board of the Netherlands Cancer Institute, serving as the central ethical committee for all participating institutes. Written informed consent was obtained and the study was conducted according to the principles expressed in the Declaration of Helsinki and following institutional guidelines. The experiment was conducted at the Academic Medical Center of the University of Amsterdam and the Spinoza Centre for Neuroimaging.

### Procedures

Baseline data were collected after surgery but before the start of adjuvant treatment (T1). All breast cancer patients underwent surgery with general anesthesia. Follow-up assessments took place at approximately six months after the last cycle of chemotherapy for the BC+SYST group and at matched intervals for the BC and the NC group (T2).

For a more detailed description of the procedures see Menning et al. [12]. In brief, the assessment consisted of questionnaires, neuropsychological assessment and multimodality MRI. We additionally collected hair samples for cortisol measurements. The current report will focus on longitudinal analyses of task-related functional MRI. Analyses of neuropsychological performance are described in [16]. Findings for other MRI modalities will be described elsewhere.

Seven questionnaires were administered to assess patient-reported outcomes (PROs) on frequently experienced symptoms by cancer patients such as fatigue, emotional and cognitive changes: European Organization for Research and Treatment of Cancer (EORTC) Quality of Life Questionnaire C-30 (QLQ-C30)[17], Hopkins Symptom Checklist-25 (HSCL-25) [18], Profile of Mood States (POMS) [19], Perceived Stress Scale (PSS) [20], Trauma Screening Questionnaire [21], Medical Outcomes Study—Cognitive Functioning Scale-revised (MOS-cog) [22], Ten-Item Personality Inventory [23]. Next to the questionnaires administered at T1, the Impact of Events Scale was used to assess distress related to breast cancer at T2 [24]. A comprehensive neuropsychological test battery was used, consisting of 18 test indices (see Menning et al. [12] for a detailed description). Verbal IQ was estimated with the Dutch Adult Reading Test (NART) [25].

MRI data were acquired using a 3.0 Tesla Intera full-body MRI scanner (AMC Medical Center) and a 3.0 Tesla Achieva full-body MRI scanner (Spinoza Centre for Neuroimaging) (Philips Medical Systems, Best, The Netherlands). A SENSE 8-channel receiver head coil was used at both locations. Functional MRI acquisition was based on T2* weighted gradient echo planar imaging (EPI) of 38 axial slices (voxel size 2.3 x 2.3 x 2.3 mm, interslice gap 0 mm, matrix size 96 x 96, TR = 2.1s, TE = 25ms). We acquired 230 volumes during the Tower of London (ToL) task, 170 for memory encoding, and 125 for memory retrieval. A T1 weighted three-dimensional magnetization prepared rapid gradient echo (MPRAGE) scan (TR/TE = 6.6 / 3.0 ms, FOV 270 × 252, 170 slices, voxel size 1.05 ×1.05 × 1.20 mm) was made for spatial normalization of the fMRI scans and anatomical reference.

**Fig 1:**
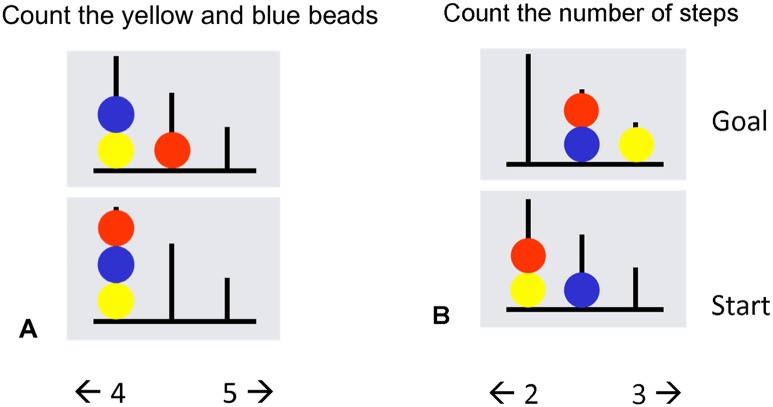
Tower of London (ToL). (A) Baseline condition; (B) planning condition. (A) In the baseline condition, participants had to count to total number of yellow and blue beads. (B) During the planning condition, participants were instructed to count the minimum number of steps required to get from the start to the final target configuration. In both conditions, two response options were displayed on the bottom of the screen (range: 1–5).

An abbreviated version of the Tower of London (ToL) paradigm by van den Heuvel et al. [26] (Fig 1) was used to assess prefrontal function. During planning, five conditions ranging from one to five moves were presented. A starting configuration and a target configuration were displayed. Each consisted of three colored beads placed on three vertical rods, which could accommodate one, two and three beads respectively. Participants were instructed to determine the minimum numbers of steps required to get from the starting to the target configuration by mentally moving the beads one at a time. In the baseline condition, the number of yellow and blue beads had to be counted. Two response options were displayed on the left and right side of the screen corresponding to either response button. The correct answer ranged from one to five steps. The presentation of trials was self-paced with a maximum duration of one minute per trial. The task lasted eight minutes. Participants were instructed to focus on accuracy rather than on speed.

**Fig 2:**
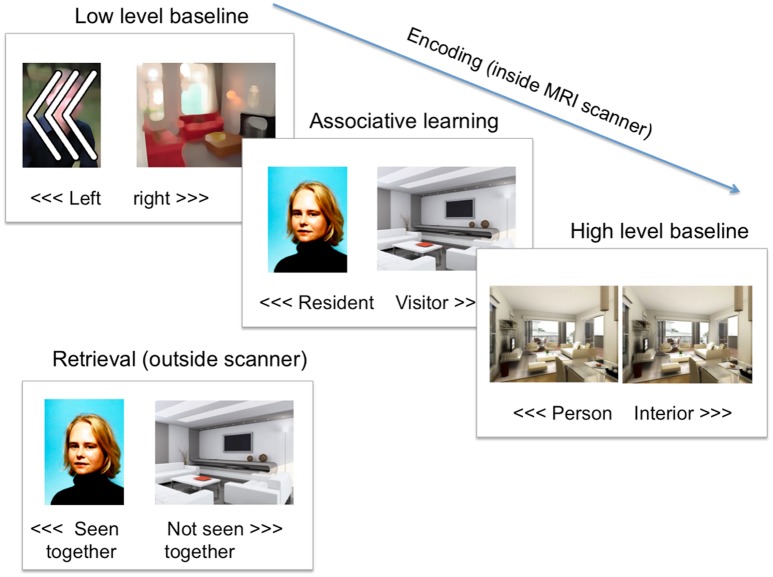
Paired associates memory task. Participants had to press a button according to the direction of the arrows (low-level baseline) or indicate whether the picture represented a person or an interior design (high-level baseline). During associative learning participants indicated whether the depicted person was likely to live in the depicted interior. During the retrieval (outside the scanner) participants indicated whether they had seen the specific stimulus pair or not.

The Paired Associates memory task was based on a task paradigm by Jager et al. and was shown to reliably activate the parahippocampal region [27] (Fig 2). During associative learning, participants were asked to indicate if the person shown in the portrait photo was likely to live in the home interior in the simultaneously presented picture. This was done by indicating “resident” or “visitor”, using a button press. The baseline condition consisted of three arrowheads pointing to the left or right, indicating a left or right button press respectively. The arrows were superimposed on blurred portrait and interior design pictures to match the visual input of the associative learning condition. The learning and baseline trials were presented in a block design. For the learning condition, six blocks were presented with five trials per block. Stimuli were presented for five seconds. The baseline condition consisted of five blocks with five stimuli, which were presented for three seconds each. Directly after the learning part of the task, a recognition test was administered. The baseline trials were the same as in the learning part. For the recognition part, all pictures from the learning phase were shown and participants were asked to indicate with a button press whether they had seen the same combination of pictures before. Sixty percent of the pairs were the same as in the learning phase. The order of the trial types was pseudo-randomized. All stimuli were presented for four seconds. Both tasks were practiced outside the scanner.

### Statistical analysis

Demographic and clinical variables, PROs and fMRI performance data were analyzed with SPSS 22 (IBM, Armonk, NY). We assessed differences between groups on PROs and fMRI performance data at T2 using univariate analysis of covariance (ANCOVA), adjusted for baseline. Reaction time (RT) for fMRI tasks was calculated for correct trials. Memory retrieval performance was calculated by subtracting the proportion of false alarms from the proportion of hits. For these analyses, the significance level was set at 0.01, instead of the more conventional 0.05. Significant overall group differences were followed by paired group comparisons.

For the ToL fMRI data, all active versus baseline trials as well as a parametric contrast with increasing task load (ToL Load) were modeled. For the ToL task load contrast, condition four and five were taken together to ensure enough trials for analysis. For the Paired Associates task, encoding trials were contrasted to baseline trials, for retrieval, hits were contrasted to baseline.

Artrepair was used to detect and repair image artifacts [28]. All fMRI preprocessing and statistical analyses were performed using SPM8 (Statistical Parametric Mapping; Wellcome Trust Centre for Neuroimaging, London UK). Slicetiming correction was applied to the images of event-related tasks, the ToL and Retrieval and not for the block designed encoding task. All fMRI images were reoriented and realigned to the first volume. Individual T1 scans were segmented based on gray matter, white matter and cerebrospinal fluid. Coregistered EPI and T1 scans were normalized to the Montreal Neurologic Institute (MNI) reference brain with use of the segmentation parameters. Finally, smoothing was applied using an 8-mm full-width half-maximum Gaussian kernel.

Individual maps of activation related to the presented tasks were analyzed using a general linear model. Main task effects were assessed using a full factorial design, group-by-time interactions were assessed using a flexible factorial design, both embedded in SPM8. Main task effects were inspected at an initial threshold of *p* < 0.001, which protects against type I errors when cluster-based thresholding is applied [29]. Whole brain group differences were subsequently considered statistically significant at a cluster-defining threshold of *p* < 0.05, corrected for multiple comparisons according to the Family Wise Error (FWE) method.

Correlations between BOLD signal extracted from regions showing group-by-time interactions and PROs were calculated separately for the patient groups. Based on previous studies we chose to calculate correlations between BOLD signal to fatigue, depressive symptoms, cognitive complaints and perceived stress. Difference scores between BOLD signal at T1 and T2 were correlated to difference scores from PROs. To determine whether baseline PROs could be predictive of change in brain activation, we also correlated baseline PROs to BOLD difference scores.

## Results

### Participant characteristics

At T1, before the start of adjuvant treatment, 32 BC+SYST, 33 BC and 38 NC were included. Details about the recruitment are described in our previous report [12]. At T2, six months post-chemotherapy, or at a similar interval, five NCs dropped out because of personal reasons including ‘illness in the family’ and ‘no time’. Our original study plan also involved an analysis to examine the contribution of endocrine therapy on cognition. Due to the unexpected large imbalance in endocrine treatment between and within the two breast cancer groups (BC+SYST, 71%; BC, 27%), this was not feasible. Therefore, we excluded nine BC patients to create a group not receiving any systemic treatment. At T2, four BC+SYSTs and one NC declined to undergo the MRI scans because they were too anxious, and task fMRI data were corrupt for one NC, resulting in a final sample of 28 BC+SYST, 24 BC and 31 NC.

No significant difference in age was found. Estimated verbal IQ was higher in NC than the patient groups, but this difference did not reach significance (p = .073) (Table 1). A marginally significant group difference in time between T1 and T2 was found, indicating a longer period between measurements in the NC group (p = .054). At T1, 39%, 54% and 55% of the BC+SYST, BC and NC participants respectively, were postmenopausal. In the BC+SYST group all premenopausal women became postmenopausal following treatment.

**Table 1:** Subject and treatment characteristics.

	BC+SYST	BC	NC	p
	(n = 28)	(n = 24)	(n = 31)	
Age at T1 (years)	49.4 (8.8)	51.2 (6.8)	51.2 (8.2)	.649
Estimated IQ (NART)	100.7 (13.3)	104.0 (13.3)	108.4 (11.4)	.073
Education level (n(%))				
Low	0	0	0	NA
Middle	4 (14)	3 (13)	0	
High	24 (86)	21 (87)	31 (100)	
Interval T1-T2 (days)	329 (69)	341 (32)	364 (57)	.054
Scan location at FU (n)	19/9	21/3	16/15	.019
Postmenopausal (n(%))				
T1	11 (39)	13 (54)	17 (55)	.421
T2	28 (100)	14 (58)	17 (55)	.001
Lifetime estrogen exposure (yrs)	31.9 (6.0)	34.0 (5.9)	33.1 (6.1)	.441
Breast cancer stage (n (%))				
0	0	13 (54)		
1	16 (57)	11 (46)		
2	11 (39)	0		
3	1 (4)	0		
Treatment (n(%))				
Radiotherapy	23 (82)	16 (67)		
Tamoxifen	19 (68)	NA		
Chemotherapy regimen (n(%))				
AC^1^	2 (7)			
AC—docetaxel^2^	20 (71)			
AC—paclitaxel^3^	3 (11)			
FEC^4^	3 (11)			
Days since chemotherapy	201 (69)			

Values indicate mean ± SD unless indicated otherwise. BC+SYST, BC patients receiving systemic treatment; BC, BC patients not requiring systemic treatment; NC, no-cancer controls. Scan location at FU depicts number of participants at the two scan locations. Lifetime estrogen exposure was calculated by subtracting age at menarche from the age at menopause or current age, for each pregnancy an additional 0.75 year was subtracted [4]. AC = doxorubicin (Adriamycin), cyclophosphamide; FEC = 5-fluorouracil, epirubicin, cyclophosphamide.

^1^4 or 6 cycles;

^2^3 or 6 cycles;

^3^4 cycles AC followed by 4 or 12 cycles of paclitaxel;

^4^3 or 6 cycles.

Differences are considered statistically significant at a critical alpha value of 0.05.

We found significant group differences at T2 adjusted for T1 in physical functioning, fatigue (QLQ-C30) and cognitive complaints (Table 2). Post hoc analyses demonstrated lower physical functioning (p = .005) in BC+SYST compared to NC, more fatigue (p < .001) in BC+SYST compared to BC and more cognitive complaints (p = .001) in BC+SYST compared to NC and BC.

**Table 2:** Patient-reported outcomes.

	T1			T2			
	BC+SYST	BC	NC	BC+SYST	BC	NC	p
	(n = 28)	(n = 24)	(n = 31)	(n = 28)	(n = 24)	(n = 31)	
QLQ-C30							
Physical functioning§	91.2 (11.9)	87.8 (12.1)	96.3 (7.7)	83.1 (16.9)*	88.6 (15.9)	96.9 (6.5)	.005
Social functioning§	78.6 (23.5)	77.8 (26.3)	100.0 (0)	78.0 (22.2)	88.9 (20.7)	98.3 (5.1)	.011
Cognitive functioning§	80.4 (24.9)	80.6 (28.1)	90.9 (14.8)	71.4 (23.1)	82.6 (17.4)	89.4 (26.1)	.052
Global quality of life¶	75.9 (18.7)	74.7 (16.2)	89.0 (10.0)	74.1 (16.6)	80.6 (23.1)	90.0 (11.7)	.104
Fatigue#	24.6 (24.4)	35.2 (26.8)	13.6 (18.1)	32.1 (25.7)**	19.4 (22.3)	13.0 (14.9)	< .001
HSCL-25	14.5 (14.2)	11.2 (11.6)	6.2 (8.0)	11.8 (13.4)	8.9 (9.2)	4.2 (5.0)	.176
PSS	24.6 (6.7)	19.9 (8.3)	18.6 (5.0)	21.8 (7.0)	19.8 (5.9)	18.1 (4.9)	.787
POMS							
Total score	18.1 (16.6)	15.5 (13.0)	8.7 (4.8)	14.0 (9.8)	9.6 (5.2)	9.2 (6.3)	.135
Fatigue subscale	2.7 (4.2)	3.5 (5.4)	1.1 (1.5)	2.4 (3.0)	1.9 (2.3)	0.6 (1.0)	.040
MOS-cog	80.3 (17.3)	72.9 (17.6)	84.4 (11.8)	71.9 (18.9)*/**	79.7 (13.0)	85.0 (10.6)	.001
IES				26.5 (11.5)	21.9 (5.9)		.072

Values indicate mean ± SD unless indicated otherwise. BC+SYST, BC patients receiving systemic treatment; BC, BC patients not requiring systemic treatment; NC, no-cancer controls; QLQ-C30, European Organization for Research and Treatment of Cancer health-related Quality-of-life Questionnaire: scores range from 0 to 100, higher score indicates § better functioning, ¶ better quality of life, or # more symptoms; HSCL-25, Hopkins Symptom Checklist-25: scores range from 0 to 100, higher score indicates higher levels of anxiety and depression; PSS, Perceived Stress Scale: scores range from 10 to 50, higher scores indicate higher levels of perceived stress; POMS, Profile of Mood States, higher scores indicate more problems; MOS-cog, Cognitive Functioning Scale of the Medical Outcomes Study, lower scores indicate more problems; IES, Impact of Event Scale, higher scores indicate more distress. P-values indicate overall group differences at T2 adjusted for scores at T1.

*Indicates a significant difference with NC at p < .01.

** Indicates a significant difference with BC at p < .01.

### Behavioral and neuroimaging results

All but three BC+SYST and one NC were scanned at the same location at both time points. The distribution of participants in each group scanned at each of the two locations differed per group (see Table 1).

No differences in BOLD signal between scan locations were found.

#### Tower of London

For the Tower of London (TOL), no fMRI data were available for one BC+SYST, whereas three BC+SYSTs, four BCs and four NCs were excluded because of artifacts, and one NC was excluded because of an error in response recording, leaving 24 BC+SYST, 20 BC and 27 NC for behavioral and fMRI analysis.

Performance and mean reaction time at T2 adjusted for T1 on the TOL task were not significantly different between groups (Table 3).

**Table 3:** Tower of London and paired associates task performance, fMRI results.

**Tower of London**									
**Task performance**									
		T1			T2				
		BC+SYST	BC	NC	BC+SYST	BC	NC	p*	
		(n = 24)	(n = 20)	(n = 27)	(n = 24)	(n = 20)	(n = 27)		
Mean proportion correct		0.88 (0.10)	0.86 (0.09)	0.87 (0.11)	0.89 (0.09)	0.87 (0.10)	0.87 (0.11)	.767	
Mean reaction time (s)		10.32 (2.80)	10.35 (3.22)	9.64 (2.25)	9.86 (2.57)	10.45 (3.87)	9.05 (2.77)	.424	
**fMRI Group-by-time interactions**									
**TOL Task Load**									
Whole brain									
	Region	R/L	MNI coordinates			Cluster (k)	*t* val.	*z* val.	p**
			x	y	z				
BC+SYST>BC	IPC / occipital cortex	l	-18	-64	36	298	5.29	4.82	.002
			-26	-74	36				
			-44	-72	18				
		r	20	-64	36	526	4.57	4.25	< .001
			34	-40	38				
			8	-60	38				
	Precuneus / superior parietal cortex	l	-2	-50	58	316	4.51	4.20	.001
			-10	-64	58				
			-14	-72	56				
BC<NC	Inferior parietal cortex	r	36	-40	38	149	3.99	3.77	.047
			30	-42	38				
			34	-40	50				
**Paired Associates task**									
**Task performance**									
		T1			T2				
		BC+SYST	BC	NC	BC+SYST	BC	NC	p*	
		(n = 25)	(n = 24)	(n = 30)	(n = 25)	(n = 24)	(n = 30)		
**Memory encoding**									
Mean reaction time (s)		2.79 (0.42)	2.87 (0.37)	2.92 (0.44)	2.72 (0.52)	2.87 (0.40)	2.78 (0.41)	.464	
		BC+SYST	BC	NC	BC+SYST	BC	NC	p*	
		(n = 25)	(n = 22)	(n = 29)	(n = 25)	(n = 22)	(n = 29)		
**Memory retrieval**									
Net performance		0.50 (0.18)	0.48 (0.19)	0.51 (0.18)	0.52 (0.17)	0.49 (0.16)	0.53 (0.14)	.743	
Mean reaction time (s)		2.25 (0.21)	2.33 (0.24)	2.34 (0.27)	2.28 (0.27)	2.36 (0.26)	2.39 (0.27)	.645	

Values indicate mean ± SD; Mean reaction time in seconds. BC+SYST, BC patients receiving systemic treatment; BC, BC patients not requiring systemic treatment; NC, no-cancer controls. IPC, inferior parietal cortex.

*P-values indicate overall group differences at T2, adjusted for scores at T1,

**FWE cluster corrected p-values

**Fig 3:**
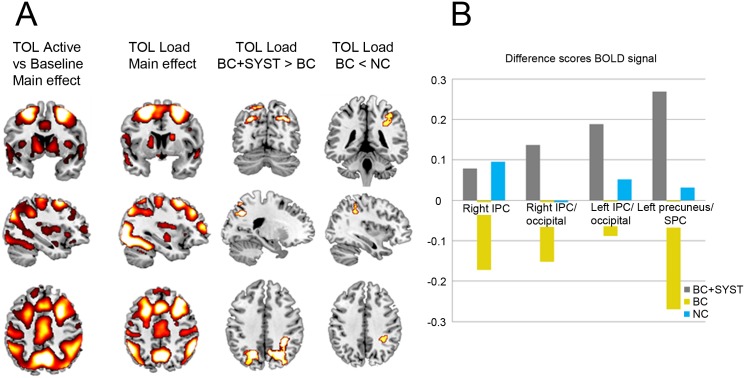
Tower of London (TOL). (A) Main task effect and group comparisons (group differences were considered statistically significant at cluster-corrected p_fwe_ < .05; brighter colors indicate higher T-values). BC+SYST, BC patients receiving systemic treatment; BC, BC patients not requiring systemic treatment; NC, no-cancer controls. (B) Difference scores of extracted BOLD signal of parietal brain regions with clusters that show significant differences between BC+SYST and BC during increasing task load of the Tower of London. IPC, inferior parietal cortex; SPC, superior parietal cortex.

For both the active versus baseline and the task load contrast, robust activation of the dorsolateral prefrontal cortex (DLPFC), premotor cortex, precuneus, posterior parietal cortex (PPC), striatum and cerebellum was found in all groups (Fig 3A).

Using a flexible factorial design, no significant differences between groups over time were found in the BOLD signal when planning trials were modeled against baseline trials.

Whole-brain analyses, demonstrated increased activation in the bilateral inferior parietal cortex (IPC) and the precuneus extending into the superior parietal cortex (SPC) with increasing task load for BC+SYST compared to BC (Table 3, Fig 3A). Activation in the right IPC decreased over time in BC compared to NC with increasing task load. BOLD signal was extracted from clusters where significant group-by-time interactions were identified.

The extracted BOLD signal showed that activation increased in several parietal clusters in the BC+SYST group with increasing task load during the TOL. In the BC group, BOLD signal in these clusters decreased over time, while the NC group showed very little change over time (Fig 3B).

#### Memory encoding

For memory encoding, fMRI data were missing for two BC+SYST. In addition, one BC+SYST and two NCs were excluded because of artifacts. fMRI memory encoding analyses were therefore performed with 25 BC+SYST, 24 BC and 30 NC.

**Fig 4:**
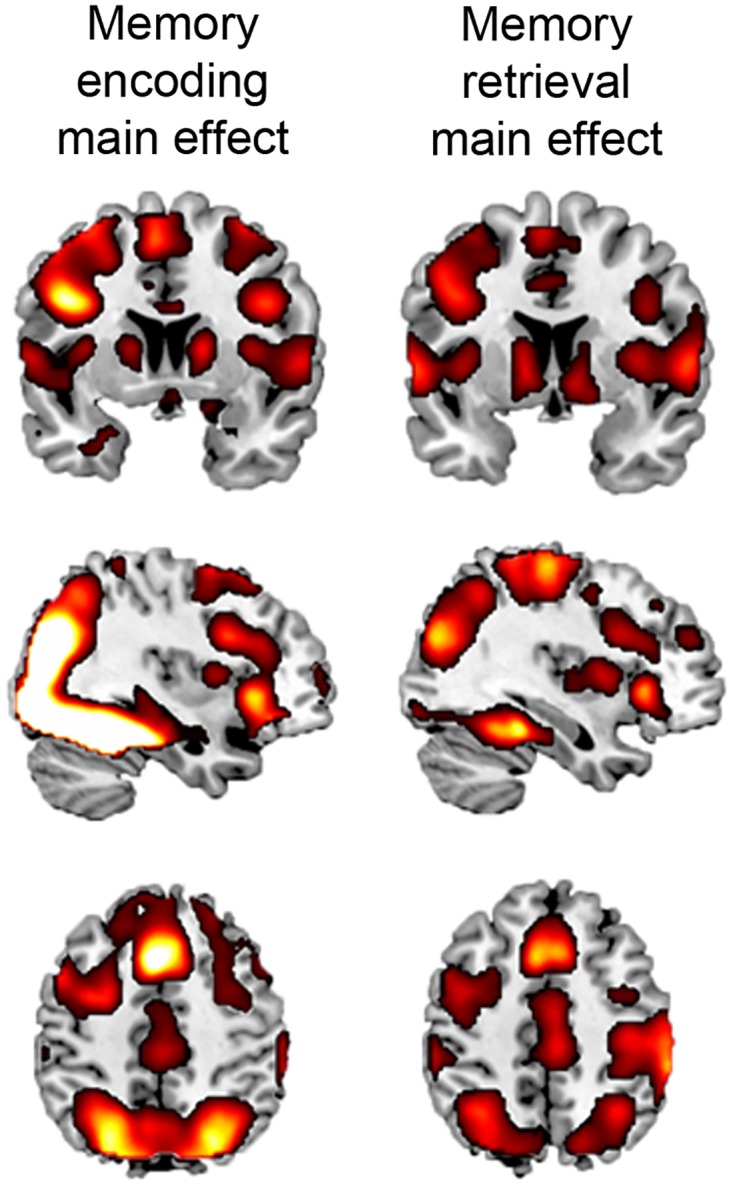
Episodic memory task. Main task effects; brighter colors indicate higher T-values). BC+SYST, BC patients receiving systemic treatment; BC, BC patients not requiring systemic treatment; NC, no-cancer controls.

Reaction time during memory encoding at T2, corrected for T1, was not significantly different between groups (Table 3). All groups showed robust activation of the ventral stream, including occipital areas, fusiform gyrus, extending into the hippocampal formation (Fig 4). Whole-brain analyses showed no significant differences between groups.

#### Memory retrieval

fMRI data were missing for one BC+SYST and two BCs. Further, due to an error in response recording, one BC+SYST and one NC had to be excluded for the memory retrieval analyses. An additional two NCs and one BC+SYSTs were excluded because of artifacts. Final analyses of behavioral and fMRI data for memory retrieval were performed with 25 BC+SYST, 22 BC and 29 NC.

For memory retrieval, no differences in mean reaction time and net performance were found between groups at T2 (Table 3). During memory retrieval, all groups robustly activated the dorsal and ventral stream (Fig 4). No significant group-by-time interactions were found for memory retrieval.

### Correlations

For the ToL task, the BC group showed a correlation between baseline fatigue and change in BOLD signal in the right inferior parietal cortex (IPC) (r = .49, p = .028). Also, a negative correlation was found between change in fatigue and change in BOLD signal in the right IPC in the BC group (r = -0.63, p = .003). No significant correlations were found within the BC+SYST group. No significant correlations between BOLD signal during the paired associates task and PROs were found.

## Discussion

This study aimed at prospectively investigating side effects of anthracycline-based adjuvant chemotherapy (with or without endocrine treatment) on brain functioning during cognitive performance six months after the last cycle of chemotherapy.

Following treatment, BC+SYST showed an increase in activation in parietal brain regions during performance on an executive functioning task compared to the pretreatment assessment. Although task performance remained stable, this hyperactivation was accompanied by worsening of physical functioning, higher levels of fatigue and more cognitive complaints. Hyperactivation after systemic treatment is compatible with an account of hyperactivation reflecting compensatory processes to maintain adequate levels of performance, although we did not find a direct association of an increase in BOLD activation with improved task performance in the BC+SYST group. Hyperactivation in the absence of differences in task performance has been suggested to reflect compensatory processes by other authors (e.g., [30–32]). This over-recruitment of brain regions might be interpreted as a response to decreased neural integrity as a result of neurotoxic side effects of systemic treatment [8]. In contrast, no significant group differences in brain activation during memory encoding or retrieval were found. It could be that the memory task was too difficult, reflected in a floor effect in behavioral as well as imaging data.

In the BC group that was not exposed to any systemic treatment, an opposite pattern of results emerged: whereas task performance remained stable, parietal activation decreased over time compared to the other two groups and compared to the pretreatment level. Differences compared to NCs could indicate that the cancer disease process itself also affects brain functioning. At baseline, the BC group showed worse cognitive function, higher levels of fatigue and worse quality of life, but values had returned to normal at the second assessment. Interestingly, at baseline the BC group showed significant prefrontal hyperactivation compared to NCs in the absence of differences in parietal activation [12]. Possibly, prefrontal areas are more susceptible to factors related to a stressful event such as cancer diagnosis and surgery, which in our sample may have been reflected in increased levels of fatigue. These effects disappeared at six months following treatment. Concurrent with normalization of potentially interfering prefrontal processes, recruitment of parietal areas possibly became more efficient, reflected in decreased activation over time.

Prior to treatment, we found fatigue to be related to worse cognitive performance, prefrontal hypoactivation and lower white matter integrity [12]. After treatment, we found an association between baseline fatigue as well as the change in fatigue and activation of the inferior parietal cortex during the TOL. This relation was only found in the BC group and not in the other groups. Higher levels of baseline fatigue were associated with a larger increase in parietal activation, suggesting patients who experience higher levels of fatigue at baseline to be more vulnerable to develop deviating patterns of brain activation. However, when levels of fatigue decreased over time, returning to normal values, parietal activation also increased, contradicting a direct association between fatigue and brain activation. This could indicate that levels of fatigue represent another factor playing a role in cognitive function and brain activation in BC patients. Previous studies have also suggested psychosocial and biological factors, such as worry [33], fatigue [34], stress [35], or cytokines [36] to be related to cancer-related cognitive impairment. However, none of the factors proposed has been found to be able to reliably predict or explain cancer treatment related cognitive impairment.

Previous longitudinal studies have also demonstrated differences in brain activation between BC patients and controls [9–11]. The study by McDonald et al. [9] most closely resembles the current study in terms of patient groups, study design and the executive functioning task. In that study, similar to our previous report [12], prefrontal hyperactivation was found in patients at baseline. A drop in activation was reported one month after chemotherapy [9], which we could not replicate here because we did not have a one-month post-treatment assessment. Prefrontal hyperactivation was shown to reappear and further increase in CHT+ patients at approximately one year after treatment. In the current study, differences between BC+SYST and the other groups at six months after treatment were located in the parietal cortex, not in prefrontal areas. Although the tasks in both studies relied heavily on executive functioning and both activate a common frontoparietal network, the task employed by McDonald et al. [9] was a verbal task, whereas a visuospatial task was used in the current study. It could be that the effects of cancer- and cancer treatment related factors are reflected in different brain regions, depending on specific characteristics of the task. Increased parietal activation with increasing task difficult is common for visuospatial tasks and particularly prominent for this specific version of the Tower of London [26]. Hyperactivation in parietal regions after chemotherapy might therefore specifically reflect compensatory activation of brain areas implicated in visuospatial attention Two other studies from our group used the same two tasks to study long-term effects of cancer and cancer treatment on brain activation [14,15]. These studies demonstrated significant hypoactivation in frontal and parietal areas in breast cancer patients ten years after being treated with conventional or high-dose chemotherapy. These differences in brain activation were found for the TOL as well as the memory encoding task. It could be that direct effects of systemic treatment are more specific to the cognitive domain targeted by a given task, whereas after a longer period of time, the effects become more generalized.

This study has several limitations. Inherent to this type of study, we were not able to attribute the observed effects to specific cytostatic agents. All chemotherapeutic regimens in the current study contained anthracyclins, which were recently suggested to be associated with stronger adverse effects on brain and cognition than regimens not containing anthracyclins [37]. Due to the unexpected large imbalance in endocrine treatment between the two breast cancer groups, we were also not able to perform exploratory subgroup analyses to investigate the effects of endocrine treatment on brain activation. Future studies should specifically focus on effects of endocrine treatment on cognition and brain function. As our fMRI tasks were only partly sensitive to group differences, future studies should employ fMRI paradigms that allow for detection of subtle differences in BOLD signal, as well as performance. Although the sample size of the current study was larger than previous longitudinal studies, our neuropsychological analyses showed that cognitive decline ‘only’ occurred in 16% patients who received systemic treatment [16]. This percentage is arguably too low to detect differences at the group level. Future studies might choose to include larger samples of cognitively impaired patients to allow for further investigation of biological substrate underlying cognitive impairment in BC patients.

Strengths of this study are the use of multiple task paradigms covering different cognitive domains, the longitudinal design, the low attrition rate, the inclusion of two control groups and the larger sample compared to previous longitudinal fMRI studies in BC patients.

To our knowledge, this is the first study to show that the effects of systemic treatment on brain activation depend on the cognitive task being performed. The changes in brain activation after systemic treatment may indicate decreased neural integrity.
